# Nociceptive Neurons Differentially Express Fast and Slow T-Type Ca^2+^ Currents in Different Types of Diabetic Neuropathy

**DOI:** 10.1155/2014/938235

**Published:** 2014-02-18

**Authors:** Eugen V. Khomula, Anya L. Borisyuk, Viacheslav Y. Viatchenko-Karpinski, Andrea Briede, Pavel V. Belan, Nana V. Voitenko

**Affiliations:** ^1^International Center of Molecular Physiology of National Academy of Sciences of Ukraine, 4 Bogomoletz Street., Kyiv 01024, Ukraine; ^2^State Key Laboratory of Molecular and Cellular Biology, Bogomoletz Institute of Physiology of National Academy of Sciences of Ukraine, 4 Bogomoletz Street, Kyiv 01024, Ukraine

## Abstract

T-type Ca^2+^ channels are known as important participants of nociception and their remodeling contributes to diabetes-induced alterations of pain sensation. In this work we have established that about 30% of rat nonpeptidergic thermal C-type nociceptive (NTCN) neurons of segments L4–L6 express a slow T-type Ca^2+^ current (T-current) while a fast T-current is expressed in the other 70% of these neurons. Streptozotocin-induced diabetes in young rats resulted in thermal hyperalgesia, hypoalgesia, or normalgesia 5-6 weeks after the induction. Our results show that NTCN neurons obtained from hyperalgesic animals do not express the slow T-current. Meanwhile, the fraction of neurons expressing the slow T-current did not significantly change in the hypo- and normalgesic diabetic groups. Moreover, the peak current density of fast T-current was significantly increased only in the neurons of hyperalgesic group. In contrast, the peak current density of slow T-current was significantly decreased in the hypo- and normalgesic groups. Experimental diabetes also resulted in a depolarizing shift of steady-state inactivation of fast T-current in the hyperalgesic group and slow T-current in the hypo- and normalgesic groups. We suggest that the observed changes may contribute to expression of different types of peripheral diabetic neuropathy occurring during the development of diabetes mellitus.

## 1. Introduction

Peripheral diabetic neuropathy (PDN), being one of the most frequent and troublesome complications of diabetes mellitus [1], is often accompanied with various pain syndromes [2–5]. Impairment of Ca^2+^ homeostasis [6–9] and remodeling of voltage- and ligand-gated ion channels [10–12] in nociceptive neurons under PDN have been implicated in altered nociception. Low voltage activated (LVA) T-type calcium channels (T-channels) [13], directly participating in cellular excitability as well as in intracellular calcium signaling, are crucially involved in both acute [14–19] and neuropathic pain [20–24]. It has been established that primary sensory neurons mainly express T-channels of the Ca_v_3.2 subtype [17, 25, 26]. This subtype mediates a major part of LVA Ca^2+^ current (T-current) although other T-channel subtypes are also present in these neurons and may potentially contribute to the LVA current [17, 20, 25, 27]. Moreover, C-fiber nociceptors seem to be heterogeneous regarding amplitudes, pharmacology, and biophysical properties of T-current [28–30] and might be divided into two subclasses correspondingly expressing fast or slow T-current [28]. Despite these findings, a lot of studies proving the importance of T-channels for nociception do not distinguish between C-fiber nociceptors expressing fast and slow T-currents within populations of small and medium size nociceptive neurons. Differential remodeling of fast and slow T-currents in IB_4_-positive capsaicin-sensitive small-sized DRG neurons [31], which are considered nonpeptidergic thermal C-type nociceptors (NTCN) [32], is of particular interest because of the strong involvement of these neurons in thermal pain sensitivity [33] and neuropathic pain [34, 35]. Recently it has been shown that in rats with streptozotocin- (STZ-) induced diabetes, the classical model of diabetes type 1 [4, 5], remodeling of T-channels in the NTCN neurons, was PDN type specific with substantial differences in a case of the thermal hyperalgesia *versus* norm- or hypoalgesia [27].

Here we have used rats with thermal hyper-, hypo-, and normalgesia at the same age and duration of STZ-induced diabetes to determine PDN-type-specific remodeling of T-channels underlying fast and slow LVA Ca^2+^ currents in NTCN neurons.

## 2. Materials and Methods

### 2.1. Experimental Animals

All experimental protocols were approved by the Animal Care and Use Committee of the Bogomoletz Institute of Physiology (Kyiv, Ukraine) and were in accordance with the National Institutes of Health Guide for the Care and Use of Laboratory Animals. Every effort was made to minimize animal suffering and the number of animals used.

### 2.2. Induction of Experimental Diabetes

We used a well-established model of streptozotocin (STZ) injections to induce diabetic neuropathy in young male Wistar rats (30–50 g, 21–23 days old) [5, 27]. Experimental diabetes was induced in rats by a single i.p. injection of STZ solution (80 mg/kg, i.p.). Blood glucose levels were checked on the third day after injection (to verify diabetes onset) and just before electrophysiological experiments (6-7 weeks after injections), using a blood glucometer (Accu-Chek Active; Roche Diagnostics, Indianapolis, IN, USA). Rats with values of >270 mg/dL (15 mM) were considered hyperglycemic.

### 2.3. Assessment of Thermal Nociception (Behavioral Experiments)

Nociceptive responses to thermal stimulation were measured by the Hargreaves' method [27, 36] using a paw thermal stimulation system (Plantar Test, Ugo Basile, Italy) for the measurement of paw withdrawal latency (PWL). PWL was recorded for each tested rat as a mean of 10 measurements with 5 min interval alternating left and right hind paws.

### 2.4. Preparation of Dorsal Root Ganglia (DRG) Neurons

We prepared dissociated DRG cells and used them within 6–8 h for whole-cell recordings as described previously [27]. In brief, L4 and L5 DRGs were incubated in a Tyrode's solution containing 140 mM NaCl, 4 mM KCl, 2 mM MgCl_2_, 2 mM CaCl_2_, 10 mM glucose, and 10 mM HEPES, adjusted to pH 7.4 with NaOH and supplemented with 1 mg/mL protease Type XIV (Sigma) and 0.5 mg/mL collagenase Type I (Worthington Biochemical Corporation) for 18–20 min at 35°C. Following incubation, ganglia were rinsed and dissociated by trituration with glass pipettes. Isolated neurons were plated onto an uncoated glass coverslip. All following experiments were done at room temperature.

### 2.5. IB4 Labeling and Imaging

Cells were incubated in Tyrode's solution supplemented with 10 *μ*g/mL isolectin B4 (IB4) conjugated to Alexa Fluor 568 dye (Invitrogen) in the dark for 10–12 min [27]. Cells were visualized using a standard Rhodamine Filter Set (Chroma Technology, USA) installed in TILL Photonics wide-field imaging system (TILL Photonics, Gräfelfing, Germany) based on an inverted microscope (IX71, Olympus) and containing a monochromator Polychrome V and Imago CCD camera both controlled by TILLvision software (TILL Photonics). Fluorescent images were captured via an oil immersion objective (40x UV, NA 1.35; Olympus) under standardized settings from 15 to 20 randomly selected small DRG cells on each dish before any electrophysiological recordings during the first 15 min of each experiment. The mean intensity of halo of IB4 staining around the neuronal plasma membrane was determined for each neuron. The relative intensity was calculated separately for neurons of each coverslip. The 0 and 100% intensity values for a particular coverslip were calculated by averaging the halo intensity of the two least intensely (0%) and two most intensely stained cell profiles (100%). Neurons were considered IB4 positive (IB4+) if their relative intensities exceeded 20%.

### 2.6. Electrophysiology

Electrophysiological recordings were performed using a standard whole-cell technique [27]. Electrodes were pulled from borosilicate glass microcapillaries with a filament (Sutter Instrument, Novato, CA) and had a resistance of 3 to 4 MΩ when filled with an internal solution containing (in mM) 146 CsCl, 2 MgATP, 2 MgCl_2_, 0.5 GTP-Na, 1 EGTA, 5 2Na-phosphocreatine, and 10 HEPES, adjusted to pH 7.3 with CsOH. The external solution for calcium currents recording contained (in mM) 2 CaCl_2_, 2 MgCl_2_, 158 tetraethylammonium- (TEA-) Cl, 10 glucose, and 10 HEPES adjusted to pH 7.4 with TEA-OH. Electrophysiological recordings were performed using an EPC-10/2 amplifier controlled by PatchMaster software (HEKA, Freiburg, Germany). FitMaster software (HEKA, Freiburg, Germany) was used for offline data analysis. Currents were low-pass filtered at 2–5 kHz. A T-type calcium current was evoked by step pulse to −45 mV for 500 ms after preconditioning at potential of −95 mV for 3 s. Bath application of Tyrode's solution supplemented with capsaicin (2 *μ*M) was used to test capsaicin sensitivity at the end of experimental procedure. Multiple independently controlled glass syringes served as reservoirs for a gravity-driven local perfusion system. All drugs were prepared as stock solutions: capsaicin (10 mM) in DMSO, Ni^2+^ (100 mM), and mibefradil (5 mM) in H_2_O. Drugs were freshly diluted to the appropriate concentrations at the time of experiments. All chemicals were obtained from Sigma (St. Louis, MO) unless otherwise noted.

### 2.7. Analysis

Statistical comparisons were performed using unpaired Student's *t*-test, one-way ANOVA, and Fisher's exact test. All quantitative data are expressed as means of multiple experiments ± SEM. The amplitude of T-type current was measured as a difference between the current peak value and the current value at the end of a depolarizing command pulse in order to avoid a residual HVA current. Activation and inactivation kinetics were estimated for each recorded T-type current as time constants of two-exponential fit from 10% of amplitude at rising part to the end of an evoking step. Voltage dependencies of activation and steady-state inactivation were described in a standard way [27] using single Boltzmann distributions of the following forms:

(1) $$\begin{array}{c} \text{activation}\;\;\frac{G\left(V\right)}{G_{\max}}=\frac{1}{1+\exp\left(-\left(V-V_{1/2}\right)/k\right)},\\ \text{inactivation}\;\;\frac{I\left(V\right)}{I_{\max}}=\frac{1}{1+\exp\left(\left(V-V_{1/2}\right)/k\right)}, \end{array}$$

where conductance (*G*(*V*)) was defined as PCD/(*V* − *E*_*r*_) (PCD is a *peak current density* defined as *I*_peak_/*C*_*m*_ and (*V* − *E*_*r*_) is *an electrodriving force* for a membrane potential (*V*) and a reversal potential (*E*_*r*_) obtained from interpolation of *I*(*V*) dependence); *G*_max⁡_ is the maximal conductance and *I*_max⁡_ is the maximal peak current amplitude; *V*_1/2_ is a voltage at which half of the current is activated or inactivated, and *k* represents the slope factor of voltage dependence. The fitted values for *V*_1/2_ and *k* are reported with 95% linear confidence limits.

## 3. Results

### 3.1. Different Types of PDN Induced by STZ Diabetes

Three days after diabetic induction by an injection of STZ, most (70%) rats developed strong hyperglycemia (mean glucose concentration 29 ± 2 mM) and were considered diabetic. As reported previously [27], within 6-7 weeks after injection of STZ, hyper-, hypo-, and normalgesic types of PDN were present in the population of STZ diabetic rats that was determined based on changes in a paw withdrawal latency (PWL). The animals were considered as thermally hyperalgesic if their PWL was less than 8.9 s, hypoalgesic if it was longer than 15.5 s, and normalgesic in any other cases (unchanged response, PWL within 8.9 ÷ 15.5 s) [27]. Animals with thermal hyperalgesia (*n* = 12), normalgesia (*n* = 8), and hypoalgesia (*n* = 9) were selected from the population of rats with 6-7 weeks of STZ-induced diabetes. The averaged PWLs for hyper-, hypo-, and normalgesic groups were 7.7 ± 0.3 s, 17.4 ± 0.5 s, and 12.4 ± 0.7 s, correspondingly, while the averaged PWL in control was 12.0 ± 0.7 s (*n* = 10). In agreement with the previous study [27], the blood glucose level of diabetic animals was significantly different from that of the control rats. However, no significant differences in the blood glucose level and body weight were observed between experimental rats of different diabetic groups (data not shown).

Thus, simultaneous presence of hyper-, norm-, and hypoalgesic animals was confirmed within the population of rats with 6-7 weeks of STZ-induced diabetes. These three animal groups together with control animals were further used to analyze whether there were thermal C-fiber nociceptive neurons specifically expressing fast or slow T-currents and whether there are some changes in expression of these currents associated with the different types of PDN induced by type 1 diabetes.

### 3.2. NTCN Neurons Expressing Fast or Slow T-Type Ca^2+^ Currents

**Figure 1:**
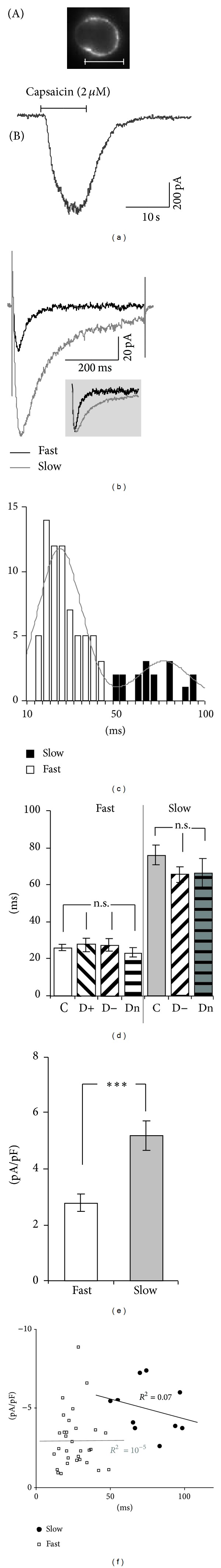
NTCN neurons express both fast and slow T-currents. (a) Identification of NTCN neurons. (A) A typical fluorescent image of an IB4-positive small-size DRG neuron. Note the intensive fluorescent ring associated with the neuronal plasma membrane. Scale bar, 20 *μ*m. (B) A typical trace of transmembrane current induced by application of capsaicin (2 *μ*M) in IB4-positive small-sized DRG neuron. IB4-positive capsaicin-sensitive small size DRG neurons were further considered as nonpeptidergic thermal C-type nociceptive (NTCN) neurons. (b) Representative current traces illustrate expression of T-currents with fast and slow kinetics of inactivation in different NTCN neurons. Currents were elicited using a 0.5 s voltage step to −45 mV after preconditioning at −95 mV for 3 s. A grey inset shows the same currents normalized by amplitude to underline a difference in kinetics of current inactivation. (c) A histogram demonstrates a pooled distribution of inactivation time constants of T-currents recorded from 85 neurons of control and PDN groups. The time constants were calculated from a single-exponential fit of current decay. A smooth curve is a fit of the distribution by a sum of two Gaussians. According to this fit T-currents were divided into fast (*τ*_in_ < 50 ms; white bars) and slow (*τ*_in_ > 50 ms; black bars) subtypes. (d) Kinetics of inactivation of fast and slow T-currents in control and PDN groups. Each column is the mean and SEM from the number of neurons specified in Figure 2(a). No significant difference compared to control was revealed under PDN conditions in kinetics of inactivation for both fast and slow T-currents. (e) Peak current density (PCD) of fast and slow T-currents under the control conditions. The columns are the mean and SEM calculated from 31 fast and 12 slow T-currents. ***P < 0.001. (f) PCD plotted *versus* inactivation time constant for fast and slow T-currents recorded under the control conditions. No significant correlations were found for both current types indicating that the difference in inactivation between fast and slow T-currents was not due to voltage clamp problems. Lines were liner fits of the dependencies; *R* ^2^ as a measure of correlation is shown in the plot.

T-current was recorded in nonpeptidergic thermal C-type nociceptors (NTCN) [32] that are strongly involved in thermal pain sensitivity [33] and neuropathic pain [34, 35]. To separate these neurons from other types of small-size DRG neurons, a population of freshly isolated cells was stained with isolectin B4 (IB4) [37] for *in vitro* labeling of nonpeptidergic neurons [32]. IB4-positive small-size neurons (Figure 1(a) (A)) were held in a voltage clamp mode at −60 mV and challenged with TRPV1 channels agonist, capsaicin. Neurons which responded to capsaicin application (2 *μ*M, 15 s) with an inward current (Figure 1(a) (B)) were considered as NTCN neurons.

T-current was recorded in these neurons using a voltage step to −45 mV after preconditioning at −95 mV. Its kinetics of inactivation (*τ*_in_) was calculated as a time constant of a single-exponential fit of decay. A strong variability of time constant values was observed within the population of neurons under study allowing suggesting that fast and slow T-current could be specifically expressed in different neurons (Figure 1(b)). A pooled distribution of *τ*_in_ values was built for T-currents recorded in neurons taken from control (*n* = 43), hyper- (*n* = 15), hypo- (*n* = 14), and normalgesic (*n* = 14) animals (Figure 1(c)) to statistically test whether different T-currents were expressed in the NTCN neurons. The distribution of *τ*_in_ values was strongly right-skewed. A Shapiro-Wilk normality test also indicated that it was not Gaussian (*P* < 2 · 10^−6^). At the same time, the distribution was reasonably fitted by two Gaussians (Figure 1(c)) suggesting the division the T-current into fast (*τ*_in_ < 50 ms) and slow (*τ*_in_ > 50 ms) subtypes. The average *τ*_in_ of the slow T-current was almost three times larger than the one of the fast T-current (Figure 1(d); Table 1). Surprisingly, in the control group a peak current density (PCD) of the slow T-current was almost twice as large as the fast one (Figure 1(e); Table 1; *P* < 0.001). Since slower decay kinetics was observed in neurons having larger PCD, this slower decay could be due to a voltage clamp problem rather than due to a difference in T-type channel gating. If this were the case, positive correlation between the T-current amplitude and kinetics of inactivation should be observed within neuronal populations expressing both fast and slow T-currents. However, no significant correlation was found between PCD and *τ*_in_ of fast and slow T-currents in the control group (Figure 1(f)), suggesting that the difference in *τ*_in_ between fast and slow T-currents was not an artifact of poor voltage clamp. Moreover, there was no significant difference in macroscopic activation kinetics between fast and slow T-currents (Table 1) which also confirmed that it is a difference in T-type channel gating that underlies fast and slow kinetics of the T-current. Thus, two groups of neurons were identified among the whole population of NTCN neurons based on their difference in T-current inactivation. It is also interesting to note that capacitance of cells expressing the slow T-current was significantly smaller (about 30%) than those expressing the fast T-current (Table 1; *P* < 0.01). This also suggests that two different neuronal groups express different T-currents.

### 3.3. Ca_v_3.2 (*α*1H) Isoform of T-Type Channels Differently Contributes to Fast and Slow T-Type Ca^2+^ Currents Expressed in NTCN Neurons

Ca_v_3.2 isoform of T-type channels is the most abundantly expressed in DRG neurons [26] and mediates the most part of T-current in NTCN neurons [27]. However, it has been reported recently that Ca_v_3.3 can also perceptibly contribute to the T-current in a subpopulation of small DRG neurons resulting in slower inactivation of the T-current these neurons express [28]. In order to examine a functional contribution of Ca_v_3.2 channels to the fast and slow T-currents in NTCN neurons of naive animals we used low micromolar concentrations of Ni^2+^ known to be a specific blocker of Ca_v_3.2 isoform with no significant effect on Ca_v_3.1 and Ca_v_3.3 T-type channel subtypes [13, 38]. A specific T-type channel blocker mibefradil [13], which is not subunit specific, was additionally used to confirm that an electrophysiologically isolated LVA current is mediated by T-type channels.

**Figure 2:**
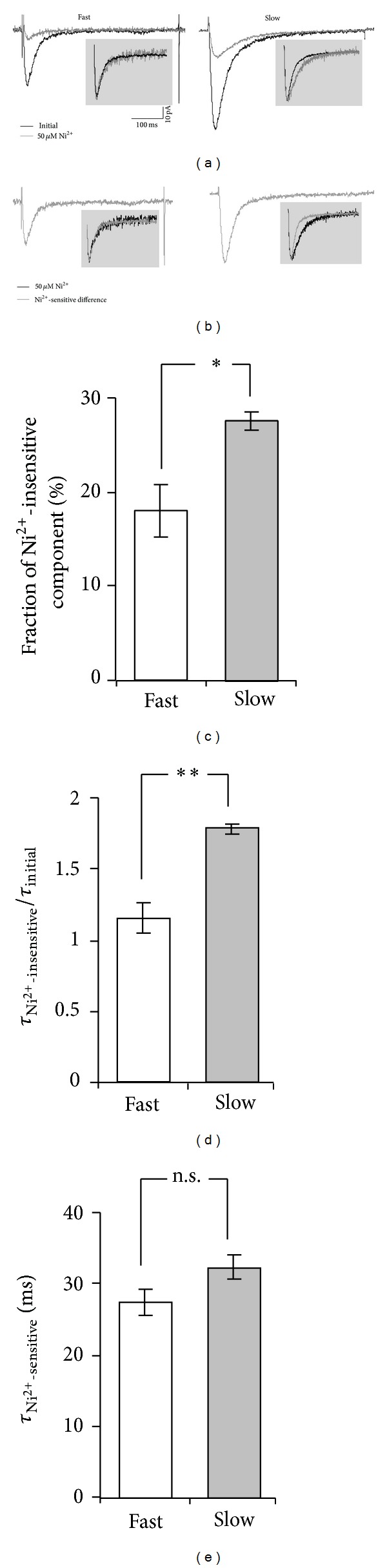
Fast and slow T-currents expressed by NTCN neurons reveal different sensitivity to low Ni^2+^ concentration. (a) Representative current traces illustrate effect of Ni^2+^ application to NTCN neurons of naive rats expressing fast (left) and slow (right) T-currents. Initial (total) T-current traces are shown in black while grey traces represent a residual Ni^2+^-insensitive component of T-current persisted during Ni^2+^ application. Note the considerably larger blocking effect of Ni^2+^ application on the fast compared to slow T-currents. Insets show the total and Ni^2+^-insensitive currents normalized by their amplitudes in order to directly compare their inactivation kinetics further shown in (c). Note the slower inactivation of Ni^2+^-insensitive component compared to the total current for the case of slow T-current. Scale bars shown in (a) are applicable to all current traces in (a) and (b). (b) Representative traces for a Ni^2+^-sensitive component of fast (left) and slow (right) T-currents were obtained by digital subtraction of the Ni^2+^-insensitive component from the total T-current for traces shown in (a). Insets demonstrate normalized Ni^2+^-sensitive (gray) and Ni^2+^-insensitive (black) components. Note the absence of visible difference in kinetics of inactivation between these components of the fast T-current and a substantially slower Ni^2+^-insensitive component as compared to the Ni^2+^-sensitive one for the case of slow T-current. (c) Fractions of Ni^2+^-insensitive component in the fast and slow T-currents were significantly different. *-*P* < 0.05. (d) A ratio of inactivation kinetics of Ni^2+^-insensitive component and the total T-current for NTCN neurons expressing the fast and slow T-currents. There were no significant changes observed in the case of fast T-current (*P* > 0.4), while the inactivation kinetics of Ni^2+^-insensitive component of slow T-current was significantly slower compared to the inactivation kinetics of the total current. **-*P* < 0.01. (e) Inactivation kinetics of Ni^2+^-sensitive components of fast and slow T-currents. n.s.: no significant difference was revealed between the inactivation kinetics of Ni^2+^-sensitive components of the fast and slow T-currents (*P* > 0.3). Each column in (c), (d), and (e) is the mean and SEM from 6 fast and 3 slow T-currents.

As expected, Ni^2+^ at low micromolar concentration (50 *μ*M) significantly and reversibly blocked both fast and slow T-currents (*P* < 0.001, Figure 2(a)). At the same time the fast T-current was significantly more sensitive to Ni^2+^ than the slow one (18 ± 3% (*n* = 6) compared to 28 ± 1% (*n* = 3) of initial current persisted for the fast and slow T-currents, resp.) (*P* < 0.02, Figure 2(c)). This finding points to a significantly larger contribution of Ca_v_3.2 isoform to the fast than to the slow T-current. There was no significant difference in kinetics of inactivation between Ni^2+^-sensitive (27 ± 2 ms) and Ni^2+^-insensitive (30 ± 2 ms) components of fast T-current as well as the fast T-current itself (26 ± 2 ms; ANOVA, *P* = 0.6; insets in Figures 2(a) and 2(b)). It is interesting to note that kinetics of inactivation of Ni^2+^-sensitive component of slow T-current (32 ± 2 ms) was not significantly different from those of Ni^2+^-sensitive component of fast T-current and the fast T-current itself (ANOVA, *P* > 0.3; Figures 2(b) and 2(e)), suggesting that this component is also mediated by Ca_v_3.2 isoform. In contrast, kinetics of inactivation of Ni^2+^-insensitive component of slow T-current (Figure 2(a), right inset) was 1.78 ± 0.03-fold slower than one of the total slow T-current. This ratio was significantly different compared to the respective ratio calculated for the fast T-current (1.15 ± 0.10; *P* < 0.01; Figure 2(d)). In addition, for the slow T-current, Ni^2+^-insensitive component was substantially and significantly slower (2.69 ± 0.12 times) than the Ni^2+^-sensitive one (*P* < 0.001; Figure 2(b), right inset), suggesting that these components are mediated by different T-channel isoforms. Altogether these results confirm our earlier suggestion about a significantly larger contribution of Ca_v_3.2 isoform to the fast than to the slow T-current. Finally, mibefradil (10 *μ*M) blocked 93 ± 3% and 93 ± 1% of fast and slow T-currents, respectively. The effect was significant (*P* < 0.001) with no significant difference between the fast and slow T-currents (*P* = 0.95), thus providing additional pharmacological confirmation that recorded fast and slow currents were mediated by T-channels.

Taken together these findings demonstrate significantly different pharmacological properties of T-channels mediating the fast and slow T-currents in NTCN neurons. While the major part of both the fast and slow T-currents seems to be mediated by Ca_v_3.2 T-type channel isoform, a considerable contribution of the other T-channel isoforms was established for the case of slow T-current.

### 3.4. PDN-Specific Functional Expression of Fast and Slow T-Currents in NTCN Neurons

**Figure 3:**
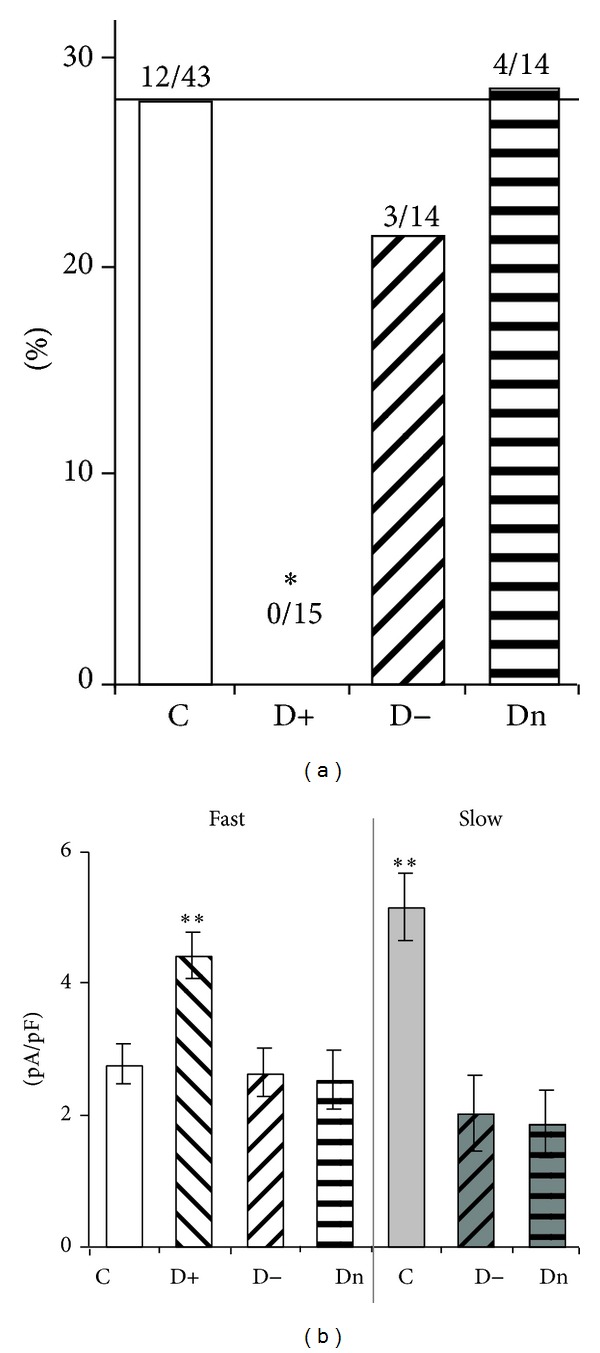
Functional expression of fast and slow T-currents in NTCN neurons under different PDN conditions. (a) Percentage of NTCN neurons revealing the slow T-current in control (C), hyper- (D+), hypo- (D−), and normalgesic (Dn) groups. The slow T-current was not observed under hyperalgesic conditions, *P < 0.05 (Fisher's exact test). The numbers above the columns indicate the number of NTCN neurons expressing the slow T-current of the total number of tested neurons in the respective group. (b) PCD of fast and slow T-currents under the control and PDN conditions. It is interesting to note that the fast T-current was upregulated in hyperalgesic conditions while the slow T-current was strongly downregulated in norm- and hypoalgesia. Each column was the mean and SEM from number of neurons specified in (a). **P < 0.01 (ANOVA). n.s.: not significant (ANOVA).

In the next phase of our research, we determined whether the NTCN neurons with the slow T-current were present under diabetic conditions and whether their percentage was differentially changed in animals with different types of PDN. We demonstrated that NTCN neurons of hypo- and normalgesic animals expressed either slow or fast T-currents, as was observed in control conditions (Figure 3(a)). The slow T-current was found in about 30% of the neurons in each of these diabetic groups with no significant differences in their fraction between both these groups and control (*P* > 0.05, Fisher's exact test). This suggests that the distribution of slow and fast T-currents among NTCN neurons was not affected in hypo- and normalgesic PDN. In contrast, the NTCN neurons of hyperalgesic rats only expressed the fast T-current (15 of 15 tested cells; Figure 3(a)). The percentage of NTCN neurons expressing the slow T-current differed significantly between hyperalgesic and control groups (*P* < 0.05, Fisher's exact test), suggesting specific abolishment of the slow T-current or elimination of the respective NTCN neurons under hyperalgesic PDN. Under diabetic conditions we did not observe a difference in capacitance between NTCN neurons expressing the slow and fast T-currents in the hypoalgesic group. However, a significant difference in capacitance was preserved in normalgesic group (Table 1; *P* < 0.01) as was initially established for the neurons of the control group.

In addition, we analyzed possible effects of differential diabetes development under different PDN on functional expression of fast and slow T-currents. We calculated the PCD of fast and slow T-currents and compared these pairwise between control, hyper-, hypo-, and normalgesic animal groups. It was found that PCD of fast T-current was substantially and significantly increased only in the hyperalgesic group (by 60 ± 20%) compared with the control group, while no significant changes were observed in hypo- and normalgesic diabetic groups (Figure 3(b); Table 1). At the same time, the slow T-current, absent in the NTCN neurons under hyperalgesia, was significantly decreased in the hypo- (by 61 ± 12%) and normalgesic (by 64 ± 10%) groups compared with the control group (Figure 3(b); Table 1). It is interesting to note that the PCD of slow and fast T-currents, which were significantly different in the control group, did not differ in the neurons of hypo- and normalgesic diabetic groups (Figure 3(b); Table 1) indicating an increased relative contribution of the fast T-current in neuronal Ca^2+^ signaling.

### 3.5. PDN Alters Biophysical Properties of Fast and Slow T-Currents in NTCN Neurons

**Figure 4:**
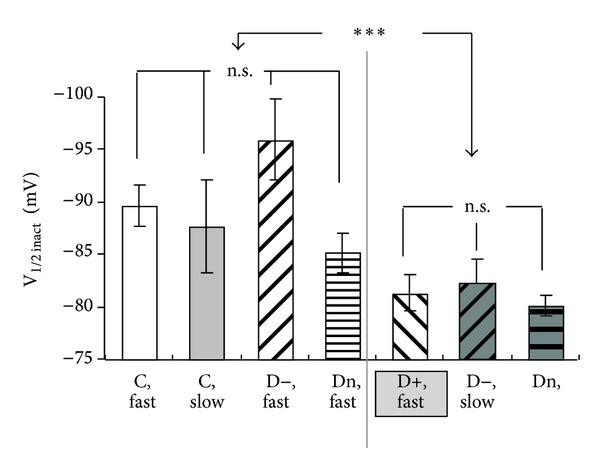
PDN-specific changes in steady-state inactivation of T-currents in NTCN neurons. Each column demonstrates the mean and SEM of half-inactivation potential of steady-state inactivation (SSI) calculated for 13 “fast” and 7 “slow” neurons of control group (C), 7 neurons of hyperalgesic group (D+), 5 “fast” and 3 “slow” neurons of hypoalgesic group (D−), and 8 “fast” and 4 “slow” neurons of normalgesic group (Dn). The results demonstrate that a depolarization shift in SSI was observed for the fast T-current in a case of hyperalgesia and for the slow T-current in norm- and hypoalgesia. ANOVA between all columns produced *P* < 0.02. ***P < 0.001 (*t*-test for merged “C, fast,” “C, slow,” and “D−, fast,” “Dn, fast” *versus* merged “D+, fast,” “D−, slow,” and “Dn, slow”). n.s.: not significant (ANOVA).

Changes in biophysical properties of T-channels may substantially influence the neuronal excitability [39]. Moreover, changes in voltage-dependent activation and steady-state inactivation (SSI) of T-channels have been recently reported for different types of PDN [27]. Therefore, the biophysical properties of fast and slow T-currents were also examined in NTCN neurons under different types of PDN (Table 2). We found that the voltage-dependent activation and SSI of fast and slow T-currents were not significantly different in control conditions (ANOVA, *P* > 0.2) (Table 2). We also determined that macroscopic activation and inactivation kinetics of the fast T-current were not significantly different between NTCN neurons of control, hyper-, hypo-, and normalgesic groups (Table 2; ANOVA, *P* > 0.3). Analogous results were obtained for the slow T-current recorded in control, hypo-, and normalgesic groups of rats (Table 2; ANOVA, *P* > 0.05). Our results suggest that activation and inactivation kinetics of fast and slow T-currents were not significantly affected under different types of PDN compared to control. No significant differences were also found in the half-activation potentials and slope factors of T-current activation and inactivation between any of the groups (control and all PDN) and in the current type (fast or slow) T-currents (Table 2; ANOVA, *P* > 0.5). At the same time, the voltage-dependence of SSI revealed a significant depolarizing shift (about 8 mV) in the half-inactivation potential of the fast T-current under hyperalgesia and of the slow T-current under hypo- and normalgesia (Figure 4) (ANOVA, *P* < 0.02), compared with the control group, Thus, activation properties of T-type channels seemed to be unaffected under PDN conditions. In contrast, SSI of slow and fast T-type currents was found to be specifically shifted in a similar way in NTCN neurons expressing slow T-current under hypo- and normalgesic PDN and fast T-current under hyperalgesic PDN.

Accordingly, we have found that two different subtypes of T-type currents distinguished by their inactivation were specifically altered under hyper-, hypo-, and normalgesic STZ-diabetic neuropathy with a prominent difference between patterns of changes observed in hyperalgesia *versus* hypo- and normalgesia.

## 4. Discussion

Recently it has been demonstrated that differences in thermal pain sensitivity between hyperalgesic, hypoalgesic, and normalgesic diabetic rats are likely due to differential changes in the functioning of TRPV1 and T-channels within a pool of NTCN neurons [27], a subclass of nonpeptidergic primary nociceptors terminating in lamina II and playing an important role in neuropathic pain [32]. In this study, functioning of T-channels in NTCN neurons was further investigated using the same experimental model of STZ-induced diabetic neuropathy. To our knowledge, this is the first study demonstrating that T-channels underlying fast and slow LVA Ca^2+^ currents are heterogeneously expressed in NTCN neurons and are specifically modulated under thermal hyper-, hypo-, and normalgesia accompanying STZ diabetes. Our results provide better understanding of potential molecular mechanisms involved in the expression of different types of PDN.

As in humans, development of PDN in rats is accompanied with various alterations in pain sensation (hyper- and, hypoalgesia, and allodynia) or leaves pain sensation unchanged (normalgesia) [2–5]. These alterations can be considered as a manifestation of different types of PDN [27]. In the current study, STZ-diabetic rats revealing different modalities of thermal nociception and simultaneously having the same age and terms of diabetes development were used as a model for the investigation of PDN-type-specific remodeling of T-type Ca^2+^ channels involved in nociception. No difference was observed in blood glucose levels and weight between rats with different modalities of thermal nociception, which suggests that the PDN-type-specific remodeling of T-type Ca^2+^ channels found in this work is unlikely due to a different metabolic state of the experimental animals and is probably directly related to a particular type of PDN.

### 4.1. NTCN Neurons Expressing Fast and Slow T-Currents

Small DRG neurons differ in the biophysical properties of expressed T-currents. About 65% of small DRG neurons express a fast inactivating T-current having biophysical and pharmacological properties resembling those of a current mediated by the T-channels of Ca_v_3.2 subtype [28, 30]. At the same time the other 35% of the neurons express a slowly inactivating T-current consisting of two pharmacologically separable components. This has been attributed to a different Ca_v_3 subunits composition expressed by these neurons [28]. Therefore, a heterogeneous population of small DRG neurons can be divided into two classes [28] that can be in particular distinguished by a rate of T-current inactivation. Differences in sensitivity to capsaicin and mechanical stimulation between these two classes of small DRG neurons [28] allowed assuming that these neurons might also be functionally different. According to our results, NTCN neurons may be also divided into two classes characterized by the expression of either a fast or slow T-current, correspondingly. The fraction of “slow” neurons (~30%) observed in our experiments is also close to the reported one for the whole population of small DRG neurons [28]. The “slow” neurons were slightly (~30%) but significantly smaller and exhibited significantly lager (~2-fold) T-currents than “fast” ones, which is also in good agreement with previous findings [28].

### 4.2. T-Type Ca^2+^ Channels Mediating Fast and Slow T-Currents

A difference in kinetics of inactivation between the fast and slow T-currents observed in this research could arise from differential expression of various isoforms of T-type channels. Indeed, two isoforms of T-type channels, Ca_v_3.2 and Ca_v_3.3, have found to be expressed in DRG neurons [26, 40]. Ca_v_3.2 is the most abundant isoform [26] both within the soma and peripheral axons of small and medium DRG neurons [41] and it mainly underlies the T-type current in small [20] (including NTCN [27]) and medium [25] DRG neurons, classically considered as nociceptive. Coste et al. have recently suggested that a proportion of small DRG neurons functionally express the Ca_v_3.3 isoform [28]. The T-currents mediated by the Ca_v_3.2 channels are known to have fast inactivation (*τ*_inact_ ~ 20 ms) while the Ca_v_3.3-mediated current exhibits substantially slower kinetics of inactivation (*τ*_inact_ ~ 70 ms) [13, 38]. These data are in a good agreement with our results in respect of inactivation time constants of fast and slow T-currents and suggest that the fast T-current might be mediated by Ca_v_3.2 channels, whereas Ca_v_3.3 channels may contribute to the slow T-current.

Previous findings indicate that blockers of Ca_v_3.2 channels substantially suppress T-currents in the whole population of small DRG neurons, including the NTCN ones [20, 27]. In this study Ni^2+^ at a low micromolar concentration also significantly blocked both fast and slow T-currents (Figure 2(a)), suggesting high contribution of Ca_v_3.2 to both of them. At the same time the fast T-current was found to be significantly more sensitive to Ni^2+^ than the slow one (Figure 2(c)). Together with the slower kinetics of inactivation of Ni^2+^-insensitive compared to Ni^2+^-sensitive component of the slow T-current (Figure 2(b), right inset) this finding additionally confirms a contribution of Ca_v_3.3 channel isoform to the slow T-current.

It was found in this study that 82% of fast T-current was blocked by 50 *μ*M of Ni^2+^. This value is close to 81% calculated for Ni^2+^-induced block of Ca_v_3.2-mediated current based upon IC_50_ = 10.3 *μ*M and *n* = 0.9 reported in [25]. It allows us to assume that the fast T-current is solely mediated by Ca_v_3.2 channel isoform (including 18% of the residual current observed in the presence of 50 *μ*M of Ni^2+^). This assumption is strongly supported by the fact that the kinetics of inactivation of the fast T-current itself as well as its Ni^2+^-sensitive and Ni^2+^-insensitive components seems to be the same (insets in Figures 2(a) and 2(b)). If the same extent of Ni^2+^-induced block of Ca_v_3.2-mediated current is present in the slow T-current then one can estimate from Figure 2(c) that Ca_v_3.3 channel isoform contributes about 12% to an amplitude of the slow T-current. This value is close to 15% observed by Coste et al. for Ca_v_3.3-mediated current in a proportion of small DRG neurons [28]. It is interesting to note that, despite a relatively low contribution to the amplitude, Ca_v_3.3 isoform accounts for about 30% of charge transferred by the slow current since kinetics of inactivation of Ca_v_3.3-mediated current is at least 2.69 times slower (Figure 2(b)). Thus, it seems very likely that coexpression of Ca_v_3.2 and Ca_v_3.3 isoforms of T-channels underlies the slow T-current in a subpopulation of NTCN neurons. At the same time, according to our and others' results [28], the NTCN neurons may hardly express the Ca_v_3.3 channel isoform alone.

A difference in kinetics of inactivation between the fast and slow T-currents could be also explained by expression of Ca_v_3.2 splice variants having slower inactivation kinetics. It might be a promising hypothesis since a high contribution of Ca_v_3.2 to both fast and slow T-currents is found in this study (Figures 2(a) and 2(c)). However, the expected difference in inactivation kinetics between the Ca_v_3.2 splice variants [42] is less than found between fast and slow T-currents in this work and, therefore, could not completely account for the experimental observations. At the same time, expression of splice variants may partially contribute to the variations of kinetics observed within the fast and slow types of T-currents.

Thus, expression of various T-type channel isoforms possibly in concert with their different splice variants may account for the difference in inactivation kinetics between the fast and slow T-currents found in this work. Most probably, a population of NTCN neurons consists of two different classes. One of them solely expresses Ca_v_3.2 channels mediating the fast T-current while the other one expresses a mixture of Ca_v_3.2 and Ca_v_3.3 isoforms thus far producing the slow T-current.

### 4.3. Fast and Slow T-Currents in NTCN Neurons under Different Types of PDN

Differences in functioning of T-channels in NTCN neurons have been recently proposed as an important factor resulting in different modalities of thermal nociception under STZ-induced diabetic neuropathy [27]. The importance of Ca_v_3.2 channels in peripheral nociceptive signaling was established previously, including a key role of their upregulation in hyperalgesia under STZ diabetes and chronic constrictive injury [16–25, 43–48]. Although changes in Ca_v_3.3 functional expression have not been documented under PDN, upregulation of Ca_v_3.3 was recently implicated in the sensitization of small DRG neurons, which possibly underlies hyperalgesia in the model of spinal nerve injury [40].

Absence of “slow” NTCN neurons, most probably expressing both Ca_v_3.2 and Ca_v_3.3 channels, under hyperalgesic PDN seems to be the very interesting finding of this work. It is hardly related to diabetes-induced death of these neurons since, to the best of our knowledge, no substantial damage of small DRG neurons has been reported at 6-7 weeks of STZ-induced diabetes. At the same time, diabetes-induced upregulation of Ca_v_3.2 channels simultaneously with downregulation of Ca_v_3.3 channels may substantially accelerate inactivation of the total T-current. In this case, NTCN neurons still expressing a mixed set of Ca_v_3 channels could be classified as “fast.” This seems to be a quite realistic scenario since significant upregulation of the Ca_v_3.2 channels is a common feature of hyperalgesic PDN [20, 25, 27, 45]. Our findings of a significant increase of T-current in “fast” (presumably Ca_v_3.2 expressing) NTCN neurons under hyperalgesic conditions and a significant decrease of T-current in “slow” NTCN neurons under norm- and hypoalgesic conditions also support this explanation. Ca_v_3.3 downregulation could be, in general, a common feature of all types of PDN observed in this study. This is supported by our findings of the absence of slow T-current in hyperalgesic animals and its significant decrease in hypo- and normalgesic rats. It is interesting to note that unchanged kinetics of slow T-current inactivation together with its decrease in hypo- and normalgesic rats suggests downregulation of both Ca_v_3.3 and Ca_v_3.2 channels in the neurons expressing the slow T-current without changes in a ratio between Ca_v_3.2 and Ca_v_3.3 channels. At the same time, no significant changes in the T-current were observed in “fast” NTCN neurons of hypo- and normalgesic rats, suggesting different sensitivity of T-channel modulation in “fast” and “slow” NTCN neurons during the progress of STZ diabetes.

Another interesting finding of the current study is a depolarizing shift in voltage dependence of inactivation under STZ-induced diabetes. A similar shift in a half-inactivation potential was also reported in our previous study [27]. The novel finding of the current study is that the shift was observed only in the “fast” NTCN neurons of hyperalgesic rats and “slow” NTCN neurons of hypo- and normalgesic rats. The shift may contribute to an increase in neuronal excitability [39] of NTCN neurons possibly underlying thermal hyperalgesia under STZ diabetes [27].

Functional expression of different Ca_v_3 channels may influence Ca^2+^ signaling associated with different neuronal activities due to specific biophysical properties of these channels. Ca_v_3.2 channels were shown to be underlying after depolarization potentials and participating in rebound discharge in medium [25] and T-rich [49] DRG neurons. However, relatively fast inactivation and slow recovery from inactivation limit the ability of Ca_v_3.2 channels to respond to high frequency stimulation (>20 Hz). The role of Ca_v_3.3 channels was not profoundly studied in peripheral sensory neurons. At the same time it was shown that slowly inactivating Ca_v_3.3 channels can still contribute to Ca^2+^ entry during high frequency bursts (100 Hz) and slow, prolonged, or repetitive stimulations [50, 51] when Ca_v_3.2 channels already became inactive. It seems reasonable to assume that, in “slow” NTCN neurons, Ca_v_3.3 channels play similar role, being a sensor of high frequency bursting and slow, prolonged, or repetitive nociceptive input, thus mediating Ca^2+^ entry in response to such activities. Taken together, these considerations allow us to suggest the following functional consequences of observed changes in T-channels functioning. An almost 2-fold increase in T-channel PCD together with the shift of its half-inactivation potential in the NTCN neurons of hyperalgesic animals may result in a considerable functional upregulation of Ca_v_3.2 channels, especially at the resting membrane potential [27]. As a consequence, Ca^2+^ entry in response to each action potential and a probability of rebound discharges are increased, thus far sensitizing the NTCN neurons and contributing to the thermal hyperalgesia. In contrast, decreased functional expression of both Ca_v_3.2 and Ca_v_3.3 channels in the “slow” NTCN neurons of hypo- and normalgesic animals reduces neuronal excitability and Ca^2+^ entry contributing to the diminished pain sensation.

## 5. Conclusions

Our results demonstrate that diabetes-induced alterations in functioning of T-channels are different in the NTCN neurons expressing fast and slow T-currents and are specifically associated with different types of PDN that may underlie the variety of pain syndromes induced by type 1 diabetes.

## Figures and Tables

**Table 1:** Parameters of fast and slow T-currents in NTCN neurons.

Control		*C*, pF		T-current parameters at −45 mV					
				Peak current density, pA/pF		Time constant of activation, ms		Time constant of inactivation, ms	
		Fast	Slow	Fast	Slow	Fast	Slow	Fast	Slow
		18.8 ± 1.0	12.8 ± 1.5	2.8 ± 0.3	5.2 ± 0.5	6.6 ± 0.6	7.2 ± 0.8	26 ± 2	76 ± 5
Diabetes	Hyperalgesia	15.5 ± 1.4		4.4 ± 0.4		5.0 ± 0.9		27 ± 4	
	Hypoalgesia	17.9 ± 1.0	17.7 ± 1.0	2.6 ± 0.4	2.0 ± 0.6	7.7 ± 1.6	6 ± 1	27 ± 3	66 ± 4
	Normalgesia	2.6 ± 2.2	13.3 ± 1.0	2.5 ± 0.5	1.9 ± 0.5	6.1 ± 0.7	3.2 ± 0.3	23 ± 2	66 ± 8

**Table 2:** Parameters of T-current activation and steady-state inactivation.

Control		Steady-state inactivation				Activation			
		*V*_1/2_, mV		*k*, mV		*V*_1/2_, mV		*k*, mV	
		Fast	Slow	Fast	Slow	Fast	Slow	Fast	Slow
		−90 ± 2	−88 ± 4	5.9 ± 0.6	5.7 ± 0.7	−49.3 ± 1.3	−46 ± 2	5.1 ± 0.7	5.7 ± 1.2
Diabetes	Hyperalgesia	−81.3 ± 1.7		6.0 ± 0.2		−48.9 ± 1.6		5.2 ± 0.3	
	Hypoalgesia	−96 ± 4	−82 ± 2	4.3 ± 1.6	5.3 ± 0.5	−50 ± 4	−49.9 ± 1.7	7 ± 3	4.1 ± 0.8
	Normalgesia	−85 ± 2	−80.1 ± 1.0	5.6 ± 0.4	6.4 ± 0.2	−51.7 ± 1.4	−49 ± 2	6.3 ± 0.9	5.9 ± 0.8
